# Game changer—talent transfer pathways in sport: a systematic review

**DOI:** 10.3389/fspor.2024.1401409

**Published:** 2024-05-09

**Authors:** Adeline Green, David Fleischman, Rory Mulcahy, Bridie Kean

**Affiliations:** ^1^ School of Health and Behavioural Sciences, University of the Sunshine Coast, Sippy Downs, QLD, Australia; ^2^ School of Business and Creative Industries, University of the Sunshine Coast, Sippy Downs, QLD, Australia

**Keywords:** talent transfer, talent identification, talent development, high-performance sport, sport pathway

## Abstract

**Research question:**

Talent transfer, an accelerated sport pathway to expertise, holds considerable appeal for sports organisations. As an emerging area of academic research across a range of sport disciplines, there is opportunity for to advance knowledge and practice. This review aimed to (a) explore how talent transfer has been defined, to develop a synthesised definition; (b) systematically identify the factors that influence talent transfer; and (c) investigate how theory underpins and enhances understanding of talent transfer.

**Research methods:**

A systematic review was conducted of 12 peer-reviewed journal articles on talent transfer using the PRISMA approach.

**Results and findings:**

Aiming for a comprehensive, multidisciplinary perspective, the results: introduce a clear, synthesised conceptualisation of talent transfer as an accelerated sport pathway in which a highly trained athlete in one sport (donor sport), transitions to a new sport (recipient sport) with the potential of achieving high-performance success; systematically map influencing factors; and outline considered theories. Factors influencing talent transfer span individual, task-related, and environmental constraints. The review exposes a scarcity of theoretical foundation in current research, suggesting ecological dynamics as a promising approach to advance research and practice.

**Implications:**

Practical and theoretical implications arise, emphasising the usefulness of a synthesised definition and a multifactorial approach for designing, implementing, evaluating, and researching talent transfer pathways. This benefits sports administrators, managers, and researchers.

## 1 Introduction

International high-performance sport is becoming increasingly competitive (1). To drive high-performance sport objectives, many countries invest substantial resources into sports, including towards talent identification and development strategies and sport pathways (2). For example, UK Sport's investment in Olympic and Paralympic sports for the Paris 2024 cycle is projected to be £213 million and £62 million, respectively (3). The resources devoted by governments and sport organisations often come with the expectation of medal-winning performances. The constant push for higher levels of performance has compelled sports researchers and managers to evaluate the optimal processes of athlete identification and development, with the aim of developing successful athletes more efficiently and effectively; ultimately, improving the return on investment made to sports (1, 4).

Talent identification is the process of recognising athletes with the potential to excel in a particular sport, and talent development aims to progress the athlete towards realising their potential (1). The literature outlines distinct talent identification methods, including talent selection, when athletes already in the sport are chosen for further development opportunities, and talent detection, where individuals without extensive training histories are introduced to a new sport (5). Talent identification and development programs play an essential role within sport pathways and in the pursuit of sports excellence, by focusing on developing a smaller number of athletes who are deemed to have the greatest sporting potential (6); thereby ensuring efficient use of resources [e.g., financial, coaches; (4)]. Talent identification is based upon the premise that certain individuals possess “talent” (i.e., inherent attributes or qualities) that predispose them to excel in a particular domain or activity (7). However, the notion of talent itself is a subject of ongoing debate within the literature (7). Despite this, talent identification in practice is viewed as a necessary means to maximise the chance of success, which has led to sports attempting to identify athletes and recruit them into development programs as early as possible (8).

However, the use of early talent identification within sport systems has been criticised for being inadequate, unreliable, unethical, and having low predictive value (7, 9). Generally, talent identification is constructed on the measurement and comparison of characteristics and skills that are considered to contribute to sport performance (4). It has been argued this approach is overly simplistic, reductionist, and lacks validity—portraying talent as static (10). Additional criticisms emphasise the lack of psychological variables, and the failure to acknowledge that expertise can result from unique combinations of skills and behaviours; thus, risks deselecting athletes with potential for success (10). Further, it overlooks the impact of training and differing rates of maturation on performance (1). The latter is evidenced by studies examining the (limited) predictive value of junior sport performance for senior performance (11); and the relative age effect, where athletes born earlier in the selection year have increased selection opportunities, likely due to being more physically and psychologically developed compared to younger athletes (1). Consequently, researchers have argued for a more comprehensive, multi-dimensional talent identification approaches (12). Recently, researchers and practitioners have begun exploring an emerging talent identification method that may address some of the shortcomings of early talent identification and provide an efficient strategy to increase podium performances: *talent transfer*—when an experienced athlete transitions from their initial sport, referred to as the donor sport, to a new sport, referred to as a recipient sport (13).

Talent transfer attempts to maximise investments made toward athlete development in the donor sport by “transferring” their abilities and experiences, contributing to accelerated development in the recipient sport (14). Sporting organisations may benefit from increased chance of identifying and developing successful athletes (14), and reduced talent wastage (15). Talent transfer is supported by studies demonstrating that athletes can develop quickly and achieve elite level competition in more than one sport (16), and that senior success is positively correlated with specialisation (training and competition in one sport) at a later age, compared to early age specialization (17). Compared to other pathways, talent transfer may be associated with lower numbers of supported athletes, shorter periods of support, and higher success rates, thereby improving the return on investments by governing bodies and sports organisations (14). A recent example can be drawn from Rugby 7's, introduced at the 2016 Rio Olympics. Australia secured the gold medal by effectively implementing talent transfer pathways in their women's Rugby 7's team, targeting experienced team sport athletes to overcome the lack of established pathways and experience among athletes in Rugby 7's (18). As interest in formalised (strategic) talent transfer increases, there is a need for an evidence-base to support best practice.

Notwithstanding examples of talent transfer in research and practice, the current state of literature is emerging and fragmented, particularly relating to terminology and definitions. Numerous interchangeable terms have been used, such as mature-age talent identification, athlete recycling, and cross-over (19, 20). There is a lack of a concrete definition of what constitutes this sport pathway. Inconsistent terminology may slow research progress, hinder collective understanding, and reduce the implementation of research findings into practice. Hence, the first aim of this research was to identify talent transfer definitions in the literature and develop a synthesised definition to guide future research and practice.

Despite a lack of conceptual clarity regarding talent transfer, studies have begun to identify factors that contribute to successful implementation [e.g., (14, 21)]. An initial systematic review identified five relevant studies and established that similarities between sports underpins some transfers, but not all (22); thus, a knowledge gap regarding additional factors to consider when developing, implementing, and evaluating talent transfer pathways remains. Other studies have provided some insights on factors impacting talent transfer success, including the recipient sports' depth of international competition (14, 23); individual factors for the identification transfer athletes for specific sports (14, 23), and the role of social, psychological, and environmental factors (10, 24).

Whilst this research has employed diverse perspectives [e.g., athletes, coaches, physiologists; (13, 24)], it has predominantly utilised a sport physiology or psychology lens, with one recent study adopting a managerial perspective (21). Increasingly, sport organisations are implementing formalised talent transfer initiatives, which typically involve systematic assessment and selection of experienced athletes for targeted sports (14). To optimise talent development, retention, and performance, these programs often utilise a multidisciplinary approach, incorporating coaching, equipment access, early competition exposure, financial support, and collaboration with sports science and other professionals, collectively referred to as “deliberate programming” (14). To support evidence-based design, implementation, and management of multifaceted talent transfer initiatives, a comprehensive and multi-disciplinary perspective is required. Thus, the second aim of this research was to systematically synthesise the factors impacting talent transfer. Enhanced knowledge of the factors is particularly important as it can inform sports practitioners and policymakers on areas to target to enhance talent transfer pathways, including athlete identification and development (25), and may ensure efficient use of resources and improved outcomes (26).

Another important consideration for talent transfer is understanding how (or if) theory has or should be used. The utilisation of theories, models, and frameworks can help to analyse, explain, and predict, and can be valuable in planning, implementing, and evaluating interventions (27). In sport, these tools may assist sport practitioners with focussing on what is modifiable, identifying areas for targeted change, and structuring sport pathways and the allocation of resources in a systematic and efficient manner. Thus, sport management scholars have noted the need for greater use of theory (28, 29). Within talent development literature, many theories, models, and frameworks have been proposed, contributing to enhanced understanding of talent development and sports performance (Table 1). Of these, the Foundations, Talent, Elite, Mastery framework (FTEM), a multistage talent development framework, is the only framework that explicitly considers talent transfer. Developed by Gulbin et al. (44), to account for the variability in athlete pathways, the FTEM is based upon sport performance indicators rather than chronological or biological age. A criticism of the FTEM is the focus on descriptive stages rather than the mechanisms of talent development (10, 48); however, the authors of the FTEM promote it as a framework upon which the mechanisms can be considered (44). Van Harten et al. (22) proposed a framework that situates talent transfer within time-based talent development pyramids, however, it has been argued that time-based models and pyramid frameworks do not account for diverse pathways to expertise (16, 49), and reinforce the discredited theory of deliberate practice (14). Like the FTEM, it is predicated on descriptive stages, and as such, a gap exists in terms of a theoretical approach for understanding the mechanisms of talent transfer.

**Table 1:** Theories, models & frameworks of talent development in sport.

Reference	Name	Description
Bloom (30)	Nil	Three stage model of development: 1. Early age development phase 2. Middle years development phase 3. Later years development phase Each stage has unique challenges and demands that must be overcome to achieve expertise.
Newell (31)	Newell's constraint model	Model emphasises the integration and connectedness of different constraints (individual, environmental, and task) that dynamically interact over time to affect developmental outcomes.
Bronfenbrenner (32)	Bioecological theory of human development	Development is outcome of person-environmental relationship. Environment is viewed at five levels: micro-, meso-, exo-, macro- and chrono-systems. Model includes four concepts: process, person, context, and time.
Ericsson et al. (33)	Deliberate practice	Theory emphasises expertise is the outcome of involvement in a highly structured activity, defined as deliberate practice. Individual differences even at the elite level are related to differences in deliberate practice, rather than innate ability. Expertise can be attained in any domain through sustained deliberate practice over a period of at least 10 years (10,000 h).
Simonton (34)	Nil	A two-part emergenic-epigenetic model is proposed as a mathematical formula of talent development. The model provides analysis of individual differences and of development trajectories over time.
Wylleman and Lavallee (35)	Holistic athlete career model	Integrated model of athlete development linked to age and with three other levels of development: (a) psychological, (b) psychosocial, and (c) academic-vocational
Abbott and Collins (36)	Nil	Model highlights the progression from initial involvement to expert performer through four stages: 1. Sampling 2. Specializing 3. Investment 4. Maintenance Emphasis on psycho-behaviours.
Balyi and Hamilton (37); Balyi et al. (38)	Long term athlete development	7 stages model of development in sport: 1. Active start 2. FUNdamental 3. Learning to train 4. Training to train 5. Training to compete 6. Training to win 7. Retirement The model recommends matching the prescription of training programs with biological maturation measurements instead of chronological age.
Gagné (39)	Differentiated model of giftedness and talent	Model highlights the process of transforming gifts (natural ability) into talents (expertise), which is influenced by environmental, intrapersonal, and chance catalysts. Talent is defined as being within the top 10% amongst peers within a particular field.
Henriksen et al. (40)	Athletic talent development environment model and the environmental success factors model	Athletic talent development environment model describes the micro and macro athlete talent development environment and includes athletic and non-athletic domains. Environmental Success Factors model structures factors that contribute to success of the athlete talent development environment.
Phillips et al. (12)	Dynamical systems theory	Theory proposes the developing athlete is conceptualised as non-linear, complex, dynamical neurobiological systems. Interactions between athlete and environment are unique and can influence each athlete differently, resulting in varied adaptations and patterns towards expertise.
Côté et al. (41)	Developmental model of sport participation	Three pathways of sport participation trajectories linked to time and age: 1. Sampling: recreational participation through early sampling and deliberate play (age 6–12 years) 2. Early diversification: elite performance through early sampling (age 6–12 years) and deliberate play (age 13–15 years) 3. Early specialisation: elite performance through early specialisation and deliberate practice (age 6+).
Davids et al. (42); Seifert and Davids (43)	Ecological dynamics theory	Athletes conceptualised as complex adaptive systems, and sport performance is outcome of dynamically interacting constraints (performer, task, environment) over different time scales (practice, development, performance).
Gulbin, Croser, et al. (44)	FTEM framework	Framework presents four macro-stages (Foundation, Talent, Elite, Mastery), which are then differentiated into 10 micro-stages. Whilst linear in illustration, the framework is described as inclusive of non-linear movements including talent transfer.
Wormhoudt et al. (45)	Athletic skills model	Athletic skills model illustrates the development of movement skills from multisport participation and describes five stages: 1. Basic 2. Advanced 3. Transition 4. Performance 5. Elite Athletic Skills.
Weissensteiner et al. (46)	3-dimensional athlete development model	Athlete development is a dynamic process, and performance is underpinned by individual factors that interact with chance, environmental and system factors. Dynamic model in that the relative contribution of any of these factors can change over time.
Den Hartigh et al. (47)	Dynamic network model	Talent is modelled as a potential that is developed through complex interactions of the athlete and the environment, which are individual, dynamic, and changing over time.

In contrast to staged models, ecological dynamics has been proposed as a viable theoretical framework for understanding the mechanisms of talent development, skill acquisition, and sports performance, which are the outcome of the dynamic and interactive relationship between the athlete and their environment (42, 43; Table 1). Drawing from ecological psychology and dynamical systems theory, ecological dynamics conceptualises athletes as complex adaptive systems, emphasising the continuous and complex relationship between constraints (42, 43). Constraints are any influencing factor or variable, which are classified as individual (i.e., relating to the athlete), task (i.e., the requirements of the activity or sport), and environmental [i.e., factors extrinsic to the athlete; (31, 42, 43)]. Systematic reviews have utilised this theoretical approach to provide a comprehensive overview of mechanisms underpinning talent identification and development [e.g., (50, 51)]. What is unclear is the utility such theories offer for research on mechanisms underpinning talent transfer. Thus, the final aim was to explore how theory has been used within the existing literature, and whether the ecological dynamics theoretical framework can be used in talent transfer research.

In conducting a systematic review, the intent was to synthesise and comparatively analyse findings from qualitative and quantitative research and address specific questions of the multidisciplinary and multi-method talent transfer literature (52). Systematic reviews involve a comprehensive and systematic approach to identifying, appraising, and synthesising available empirical evidence relevant to a research question (52). They serve to inform best practice, decision-making processes, and pinpoint gaps in knowledge, providing valuable guidance for future research directions (52). Thus, this systematic approach helps make sense of the existing work by providing a comprehensive and multidisciplinary perspective on talent transfer (53). Specifically, this paper: (a) explored how talent transfer has been defined, to develop a synthesised definition; (b) systematically identified the factors that influence talent transfer; and (c) investigated how theory underpins existing talent transfer research, and whether the ecological dynamics theoretical framework can enhance our understanding of talent transfer.

## 2 Method

### 2.1 Search strategy & inclusion criteria

A systematic review of the available literature was conducted using PRISMA guidelines (54, 55), as per similar systematic reviews [e.g., (11, 50)]. Four databases aligning with the topic scope were selected—SCOPUS, Web of Science, PubMed and SPORTDiscus were searched for relevant publications using keywords sport* and athlete*. These key terms were searched alongside terms “*talent transfer*”, “*talent identification*”, “*talent development*”, “*talent pathway*”, “*talent transition*” and “*multi-sport*”. Reference lists of included articles were searched for additional articles that met the inclusion criteria. Google Scholar was used as a final search for additional publications.

Articles published prior to December 2022 which met the following criteria were included: (a) peer-reviewed article; (b) full-text availability; (c) English text; and (d) contained relevant data on the concept of talent transfer in sport [two sports at a highly trained level; (56)]. Articles were excluded if they: (a) were not an empirical article (e.g., systematic review, commentary, conference abstracts); and (b) were primarily focussed on concepts other than talent transfer pathways (e.g., skill transferability, injury).

**Figure 1:**
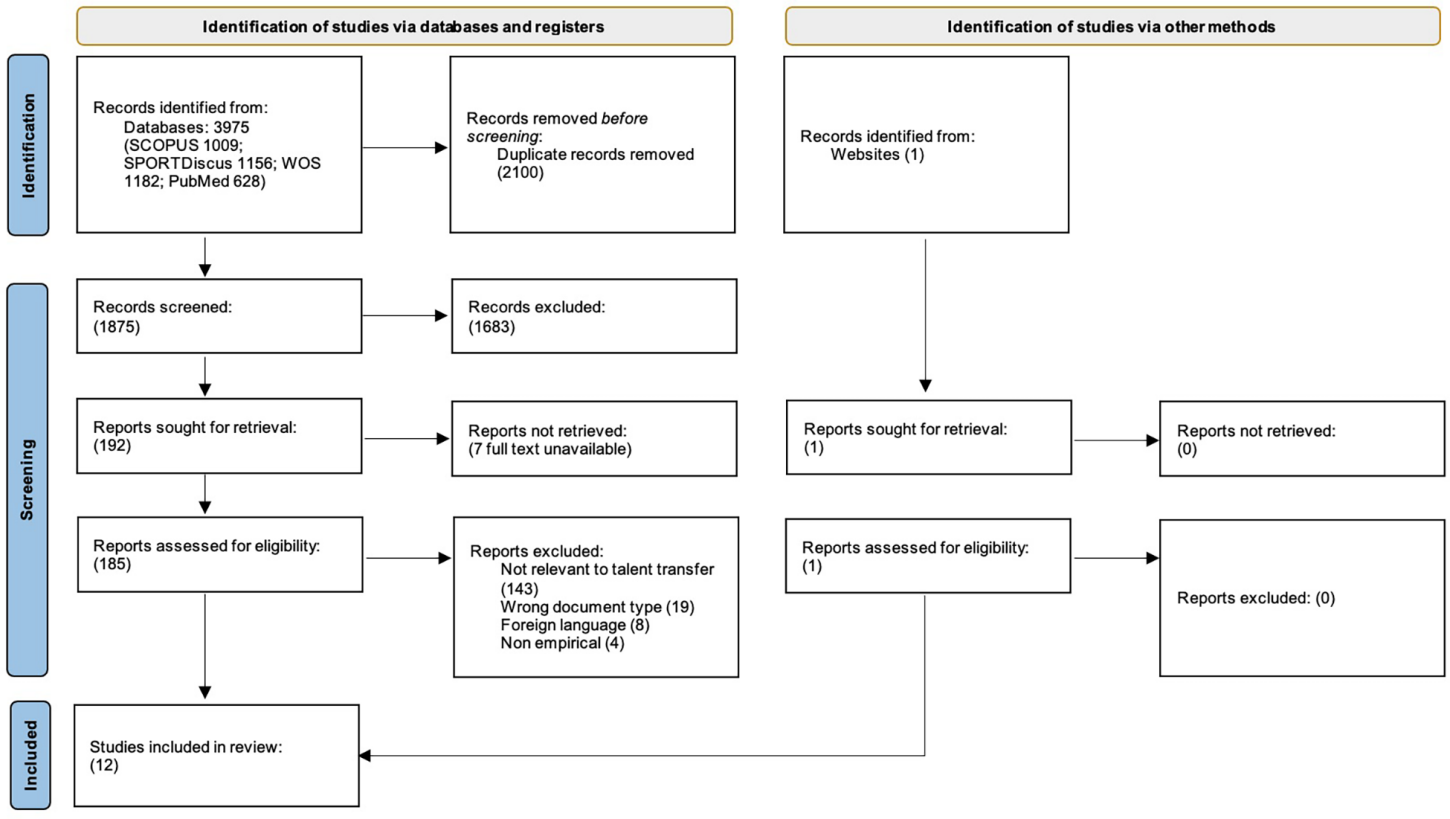
PRISMA diagram of the search strategy for peer-reviewed research on talent transfer.

The initial search identified 3,975 titles. Duplicates were removed (2,100 publications). The remaining 1,875 articles were exported to an online systematic review tool (57) and screened by two authors (AG & BK) independently for relevance based on their title and abstract, with additional 1,683 articles removed. Full texts of 192 articles were then screened to determine the eligibility based on the inclusion criteria. Any disagreement about eligibility for inclusion was resolved with a third author (DF). At the end of screening, 11 articles were retained for analysis. One additional publication was found and met the inclusion criteria during a Google Scholar search. Thus, a total of 12 publications were included for in-depth review and analysis (Figure 1).

### 2.2 Quality assessment

The methodological quality was assessed using Critical Review Forms by Law et al. (58), for quantitative studies, and Letts et al. (59) for qualitative studies, as per similar systematic reviews [e.g., (50)]. The quality assessment was conducted by two authors for the qualitative (AG & BK) and quantitative studies (AG & DF) to ensure accuracy and reliability. Each component was scored “1” (meets criteria), “0” (does not meet criteria fully), or N/A (not applicable). The final score and classification for each study was calculated following the scoring guidelines of a similar systematic review by Faber et al. (60). A final percentage score was calculated by dividing the sum of the scores of all relevant items by the total number of scored items for the specific research design, and ≤50% was considered low methodological quality, between 51% and 75% was considered good methodological quality, and >75% was excellent methodological quality (60).

The overall mean quality score for all included studies was 70.01%, with individual scores ranging from 36.04% to 93.33% (Table 2). Methodological quality was classified as: low (17%, *n* = 2), good (58%, *n* = 7), and excellent (25%, *n* = 3). Overall inter-rater reliability between the authors was calculated using the Cohen's Kappa statistic in SPSS, found to be *κ* = 0.87 and *κ* = 0.76 for the qualitative and quantitative studies, respectively, indicating acceptable agreement per the accepted classification of Landis and Koch (66).

**Table 2:** Talent transfer publications’ study design & characteristics.

Reference	Study design	Methods	Sample (Gender)	Age at transfer (years, mean ± SD)	Country	Informal/formal	Quality score (%)	Donor sport			Recipient sport			Summer/winters
								Sport	Team/individual	CGS/non-CGS	Sport	Team/individual	CGS/non-CGS	
Bullock et al. (14)	Quantitative	Anthropometric, Physical, & Performance measures; Sport specific training	10 athletes *(female)*	25.7 ± 4.6	Australia	Formal	73.33	Multiple	Individual	Both	Skeleton	Individual	CGS	Summer to winter
Collins et al. (24)	Mixed	Surveys, Interviews	Part 1: 174 athletes Part 2: 4 stakeholders (*NR)*	19	UK	N/A	36.04^a^	Multiple	Both	Both	Multiple	Both	Both	Various
Cury et al. (21)	Qualitative	Interviews	10 stakeholders (*NR)*	N/A	Australia	Formal	68.18	N/R	N/R	N/R	N/R	N/R	N/R	N/R
Hayman et al. (61)	Qualitative	Interviews	8 athletes *(4 male; 4 female)*	14.9 ± 1.0	Australia	Informal	72.73	Multiple	Both	Both	Multiple	Both	Both	Summer to summer
Hoare & Warr (23)	Quantitative	Performance measures; Sport Specific Training	24 athletes *(female)*	15.4 ± 1.06	Australia	Formal	60.00	Multiple	Both	Both	Soccer	Team	Non-CGS (Game)	Summer to summer
MacNamara & Collins (10)	Qualitative	Interviews	7 athletes *(2 male; 5 female)*	N/R	UK	Informal	72.73	Multiple	Both	Both	Multiple	Both	Both	Summer to summer
Rea & Lavallee (13)	Qualitative	Interviews	10 athletes *(4 male; 6 female)*	21.9 ± 5.2	UK	Both	90.91	Multiple	Both	Both	Multiple	Both	Both	Various
Riot et al. (62)	Qualitative	Interviews	3 athletes; 3 coaches (*NR)*	N/R	Australia	Formal	77.27	N/R	N/R	N/R	Multiple	Individual	Both	Summer to summer
Sæther et al. (63)	Qualitative	Interviews	7 coaches (*6 male; 1 female)*	N/A	Norway	Formal	72.73	Multiple	Both	CGS	Cross-country skiing	Individual	CGS	Summer to winter
Talsnes, Hetland, et al. (64)	Quantitative	Performance measures; Sport Specific Training	24 athletes *(15 males; 9 females)*	19.2 ± 1.8	Norway	Formal	93.33	Multiple	Both	CGS	Cross-country skiing	Individual	CGS	Summer to winter
Talsnes, van den Tillaar, et al. (65)	Mixed	Interviews; Performances measures; Sport Specific Training	23 athletes; 7 coaches (*14 male; 9 female athletes; coaches NR)*	19 ± 2	Norway	Formal	72.88	Multiple	Both	CGS	Cross-country skiing	Individual	CGS	Summer to winter
Teunissen et al. (19)	Quantitative	Surveys	891 coaches (*NR)*	N/A	International	N/A	50.00	Multiple	Both	Both	Canoe & kayak	Both	CGS	Summer to summer

CGS, denotes centimetres, grams, seconds; N/R, denotes not reported; N/A, denotes not applicable.

^a^ Mean of overall qualitative and quantitative quality appraisal scores.

### 2.3 Data extraction & analysis

Using Microsoft Excel, one author extracted the data, and another verified it. Data extracted included authors, year of publication, study design, method, sample characteristics (including data related to donor and recipient sports), talent transfer definition, catalyst for transfer, time to success, and other key findings. Available data relating to the donor and recipient sport were categorised and subsequently coded as: team or individual; summer or winter; and as CGS (centimetres, grams, seconds) or non-CGS [includes artistic, game, martial arts, and “other” sport; (17, 67)]. The study design, sample characteristics, and overall quality score of the included publications is illustrated in Table 2. In relation to donor and recipient sports, current research predominately focuses on individual and CGS recipient sports (Table 2).

An inductive thematic analysis of the included studies was conducted in NVivo. Thematic analysis is a theoretical and is utilised to determine patterns within a data set, and for interpreting the meaning of those patterns (68). This process involved critically engaging with the literature, coding data relevant to the research aims, and organising codes to construct categories of the recurring patterns (68). Convergent synthesis design was used, where included studies (quantitative and qualitative) were analysed using the same synthesis method, with results grouped, summarised, and presented together (69). To synthesise studies with quantitative and qualitative evidence, data transformation occurred initially to code quantitative data into themes (69).

Recurring themes, using a deductive process, were subsequently assigned to a relevant constraint: individual, task, or environmental (42, 43). Ecological dynamics theory proposes these constraints dynamically interact and impact upon skill acquisition, the development of expertise, and sports performance; thus, may provide a strong foundation for understanding the mechanisms underpinning talent transfer (43). It was therefore deemed appropriate to utilise these constraints as a theoretical lens and to group commonalities in factors across the studies accordingly. Throughout the coding process, any differences were discussed between two authors (AG & BK) and resolved to ensure consistency and validity.

## 3 Results

### 3.1 Talent transfer definition

A definition or description of talent transfer was provided in eight studies (67%; Table 3). Whilst none used identical definitions, consistent terminology and key characteristics were evident. Talent transfer was conceptualised as a process involving: high-performance or elite athletes in the donor sport (*n* = 3, 25%); the elite level in the recipient sport (*n* = 2, 17%); and accelerated development and anticipated opportunities to succeed in the recipient sport (*n* = 2, 17%). Additionally, talent transfer was described as a process facilitated by similarities in demands and skills between donor and recipient sports (*n* = 2, 17%), and the transfer of athlete's characteristics in six studies (*n* = 50%). A single study defined talent transfer from a sports management perspective, emphasising the importance of collaboration between different organisations (8%). Four studies (33%) did not explicitly provide a description or definition (Table 3).

**Table 3:** Definitions of talent transfer.

Author	Definition	Key characteristics
Bullock et al. (11, p. 398)	Existing high-performance athletes are targeted, and their athletic ability is transferred to another sport	Elite in donor sport; Athlete characteristics
Collins et al. (38, p. 3)	An athlete that had competed at international, national, or state level in both their “donor” (sport 1) and “transfer” (sport 2) sport	Elite in donor and recipient sport
Cury et al. (26, p. 11)	A structured talent transfer pathway is an accelerated talent identification and development pathway in which sports organisations (from different sports) collaborate to offer or receive talented athletes, enabling athletes to rapidly transfer to a new sport where international sporting success can be achieved typically within 3–5 years	Collaboration between sports; Accelerated developmental timeframe; Elite in recipient sport; Opportunities to succeed; Athlete characteristics
Hayman et al. (70, p. 212)	Occurs when a high performing athlete's involvement in a sport, in which they have invested significant effort and resources over substantial periods of time, comes to an end, and they try to transfer their experiences to a new sport	Elite in donor sport; Athlete characteristics
Hoare & Warr (23)	No definition supplied	N/A
MacNamara & Collins (10, p. 1)	The more or less structured transfer and fast-tracking of talented individuals from one sport to another sport where there are opportunities to succeed	Accelerated developmental timeframe; Athlete characteristics; Opportunities to succeed
Rea & Lavallee (11, p. 44)	A process occurring when an athlete ceases or reduces their involvement in a sport in which they have invested significant time, hard work, and resources, and concentrates their efforts in a sport that is new to them, but involves similar movement skills, physiological requirements, and/or tactical components of their earlier sport	Athlete characteristics; Similarities between donor and recipient sport
Riot et al. (55, p. 62)	Occurs when an athlete moves into a new sport that typically shares physical, perceptual, cognitive, and physiological requirements of their previous sport	Athlete characteristics; Similarities between donor and recipient sport
Sæther et al. (52, p. 1)	The process in which an athlete makes a change from their original sport (i.e., the donor sport) to a new sport (i.e., transfer sport)	N/A
Talsnes et al. (64)	No definition supplied	N/A
Talsnes et al. (65)	No definition supplied	N/A
Teunissen et al. (19)	No definition supplied	N/A

Taken together, these key characteristics offer some important parameters to be considered to advance the conceptualisation of talent transfer. Firstly, talent transfer should be defined in a manner that specifies the athlete was considered highly trained in their donor sport (Tier 3, national & collegiate level, in accordance with the categorisation proposed by McKay et al. (56); this differentiates talent transfer from other methods of talent identification (i.e., talent selection or detection). Additionally, definitions should specify the athlete's potential [i.e., talent; (71)] and the intention for accelerated development and success in the recipient sport. Thus, a definition that encompasses both informal and formal talent transfer is proposed:

*Talent transfer is an accelerated sport pathway in which a highly trained athlete in one sport (donor sport), transitions to a new sport (recipient sport) with the potential of high-performance success.*

### 3.2 Catalysts for talent transfer

Athletes' reasons for undertaking talent transfer were investigated in two papers (17%; Table 4). It was evident that some athletes were ambitious, and outcome focussed and transferred to pursue greater opportunities for international success (13, 61). Additionally, some athletes were unable to continue their donor sport due to a lack of a pathway to international competition (61), a plateau in athlete's performance (61), and injury (13, 61).

**Table 4:** Summary of the factors influencing talent transfer.

Reference	Catalyst	Personal				Task			Environmental					Time to success
		Anthropometric, physiological, & physical characteristics	Psychological characteristics	Age	Injury	Similarities between donor and recipient sports	Opportunities for success	Recipient sport demands	Athlete development processes	Coaching	Social support	Daily training environment	System factors	
Bullock et al. (14)		X	X	X		X	x		X	X		X		14 months
Collins et al. (24)		X	X	X		X	X	X						
Cury et al. (21)		X	X							X	X		X	
Hayman et al. (61)	X	X	X				X			X	X			<12 months
Hoare & Warr (23)		X	X				X	X	X			X		12 months
MacNamara & Collins (10)		X	X	X		X			X	X	X	X		6–24 months
Rea & Lavallee (13)	X	X	X			X			X	X	X	X	X	
Riot et al. (62)			X		X			X	X	X	X	X		
Sæther et al. (63)		X	X	X	X	X		X	X	X	X			
Talsnes, Hetland, et al. (64)		X			X				X					
Talsnes, van den Tillaar, et al. (65)		X	X		X				X	X	X	X		
Teunissen et al. (19)		X				X								

X denotes reported factor influencing talent transfer.

### 3.3 Factors underpinning talent transfer

To analyse the factors underpinning talent transfer, a constraint approach was used as a theoretical lens to analyse and synthesise the identified factors identified (42). The constructed themes relating to the factors influencing talent transfer are presented under individual, task, and environment. Upon categorisation, all included studies were multidimensional in terms of their results, which highlights talent transfer as a multifaceted process and supports the use of this theoretical approach (Table 4).

#### 3.3.1 Individual constraints

##### 3.3.1.1 Anthropometric, physiological, physical, technical characteristics

Anthropometric, physiological, and physical (i.e., technical or motor skills) characteristics were most frequently stated factors influencing talent transfer (92%, *n* = 11; Table 4). These factors were highlighted at different stages, such as during talent identification or talent development. Objective measures of these characteristics, reflecting the demands of the recipient sport, successfully identified and selected athletes for talent transfer programs (14, 23). They were also utilised to assess sport similarities to identify potential donor and recipient sports combinations (19), and to differentiate high- from low-responders in a talent transfer program (64, 65). However, three qualitative studies agreed that these characteristics alone cannot be fully explain successful talent transfer (10, 13, 24). Importantly, transfer athletes could build upon and adapt skills and characteristics developed in their donor sports, facilitating faster development compared to other athletes (10, 14, 23). The sport background of transfer athletes influenced their development in a recipient sport, with training histories in a specific donor sports having a more positive effect than others (63–65).

##### 3.3.1.2 Psychological, behavioural, and cognitive characteristics

Psychological, behavioural, and cognitive characteristics ranked as the second most frequently highlighted factor (*n* = 10, 83%; Table 4). Transfer athletes' established knowledge of high-performance sport requirements (e.g., training demands, competition, high-performance coaching, high-performance sport environments), developed in their donor sport, contributed to accelerated development (10, 14, 24). Coaches' subjectively assessed athletes' psychological and behavioural characteristics, such as their interaction with their teammates and attitudes for talent confirmation (14, 23). In a qualitative study, coaches declared motivation and resilience as the most important factor, distinguishing high- from low-responders to a talent transfer program (65). Athletes believed their previously developed psychological and behavioural strengths, such as work ethic and commitment, compensated for deficits in other areas [i.e., anthropometric, physiological; (10)]. Confidence, determination, and athlete identity were among other common facilitative psychological (10, 13, 14, 21, 24, 61–63, 65).

##### 3.3.1.3 Age

Age was discussed in four publications as an important consideration in talent transfer (33%; Table 4), but findings about the ideal age for transfer were conflicting (10, 24). Two studies suggested younger age was associated with enhanced development responses to a talent transfer program (14, 63). However, the findings were either not significant [World Cup selected athletes' mean age 22.2 ± 5.1 years, compared to non-selected athletes mean age 25.2 ± 4.6 years; (14)], or subjectively assessed (63). Interestingly, a separate publication of the same athlete cohort found age was not a significant factor when comparing high vs. low responders to a talent transfer program [18.6 ± 1.4 years and 19.7 ± 1.9 years, respectively; (63, 65)].

#### 3.3.2 Task constraints

Task constraints were found to influence talent transfer in nine studies (75%; Table 4), presented below.

##### 3.3.2.1 Similarities between donor & recipient sports

Similarities between donor and recipient sports were highlighted by six studies (50%; Table 4). Athletes transferred between sports with similar characteristics, such as motor skill and physiological requirements, believed to contribute to accelerated development (10, 13, 14, 24, 63). For example, transferring strength developed in skiing to cycling (13). Two quantitative studies explored strategic transfer between sports with similar qualities: explosive speed in beach and track sprinting and winter skeleton (14), and various other team sports to soccer (23). Another study compared sports' task constraints, based upon the notion that similarities may facilitate successful transfer (19). However, it was emphasised that not all transfers could be attributed to similarities between sports (24).

##### 3.3.2.2 Opportunities to succeed

Characteristics of the recipient sport impacted the potential for successful transfer, with some sports more feasible that others. Recipient sports with smaller talent pools or reduced depth of international competition was thought to increase the likelihood of success (14, 23, 24). For instance, Bullock et al. (14) determined there was an opportunity to transfer athletes into winter skeleton; at the time there were less than 100 registered women athletes in the sport, with less than half of them having World Cup experience. Athletes' chose to transfer into recipient sports with pathways to a professional or international level competition [e.g., the Olympic or Paralympic Games; (61)].

##### 3.3.2.3 Recipient sport demands

The complexity or technical requirements of recipient sports were important for talent transfer (*n* = 4, 33%; Table 4). Recipient sports with high technical demands or complex skill requirements may be less feasible for successful transfer, compared to less technical or complex sports (24, 62, 63). For example, a physiologist felt transferring an athlete from football, a team sport with complex skill requirements, to sprinting, an individual sport with relatively less complex skill requirements, would be more feasible than transferring an athlete from sprinting to football (24). However, one study successful transferred athletes into soccer, a team game sport, illustrating how other factors may offset the complexity of the recipient sport as a potential barrier for talent transfer [i.e., opportunities for success and similarities between sports (23)].

#### 3.3.3 Environmental constraints

The impact of environmental factors on talent transfer was discussed in 11 studies (92%; Table 4).

##### 3.3.3.1 Athlete development processes

Development processes were considered by eight studies (67%; Table 4). This included monitoring and managing training volume and load, and individualised and deliberate programming [including the strategic planning of the daily training environment, also discussed in *Daily Training Environment*; (14)]. Studies suggested transfer athletes with superior outcomes completed higher training volume and load (14, 23, 63–65), although significant differences in training hours and training volume/load ratios were demonstrated in only one study (65). Recovery and the balance between load and recovery was discussed as integral for development and performance (63). Utilising early competitions as opportunities for development was believed to accelerate development (10, 13, 14, 23, 62). Conversely, providing athletes with sufficient time to develop without early pressure for competition results was also discussed as facilitative (10, 62).

##### 3.3.3.2 High-quality and individualised coaching

Access to quality, individualised coaching was beneficial (10, 13, 14), as was positive coach-athlete relationships (21, 61–63, 65). Coaches with knowledge of the differentiated talent transfer pathway and how to accelerate the development of athletes who have minimal sport specific experience was also perceived to be advantageous (62). Similarly, one study found that coaches' lack of understanding of talent transfer was a barrier to establishing talent transfer pathways (21).

##### 3.3.3.3 Daily training environment

The daily training environment was identified as influential in talent transfer (*n* = 8, 67%; Table 4). Within the daily training environment, facilitators included: a positive, safe, and supportive learning environment; communication of feedback and the sport's expectations with the athlete; monitoring overall progress; the sporting organisation being prepared for and having previous experience with talent transfer (10, 13, 62); and supplying resources including equipment, funding, and access to sports practitioners [e.g., physiotherapy, physiology, strength and conditioning, nutrition, psychology, athlete wellbeing and engagement; (13, 14, 23, 62, 64)].

##### 3.3.3.4 System factors

Collaboration between sporting organisations was facilitative of talent transfer (21). However, fear of losing athletes, talent transfer culture, difficulty creating collaborations between sports organisations, and resourcing were barriers for establishing talent transfer pathways (21). A lack of funding or scholarships was a barrier for athletes (13). Sports practitioners believed policies and distinct frameworks (from other pathways) for providing transfer athletes with financial and performance support would facilitate faster development (21).

##### 3.3.3.5 Social factors

Social factors were discussed in eight publications (67%), and related to the athlete's family, friends, teammates, and commitments outside of sport (13, 21, 61, 62; Table 4). Athletes reported challenges adapting to a new sport environment, and no longer being “the best” at their sport (62). Athletes believed a positive support network enhanced their transfer pathway (13, 61, 62). One study found transfer athletes may not be accepted by non-talent transfer teammates, which can be a barrier in the initial stages of transfer (21), whilst formalised programs that include other transfer athletes were perceived to contribute to superior development (63, 65).

### 3.4 Use of theory

Theoretical frameworks were utilised in two papers (17%): Bullock et al. (14) utilised “Deliberate Practice” theory (33); and Riot et al. (62) utilised the “Transitions Cycle” theory (72). Most of the included publications did not explicitly apply or test a theory, model, or related elements (67%, *n* = 8). However, the Adolescent Sport Talent Transfer Stage model was developed in one study (61), and the Athletic Skills Model (45) was used to guide the design of one study (19).

## 4 Discussion

This paper addressed three aims including: identifying current definitions of talent transfer and developing a synthesised definition; developing an initial conceptualisation of factors which impact talent transfer; and exploring how theory has been used in the talent transfer literature. The findings addressing these three aims are discussed next.

An objective of this systematic review was to understand the conceptualisation of talent transfer. The findings confirm a lack of a clear, concise definition, although some similarities exist between definitions. Shortcomings included a lack of differentiation from other talent identification methods (i.e., talent detection or talent selection), and overemphasis on similarities between sports. Whilst this review demonstrate similarities between sports can be a factor, it also concludes that not all transfers can be attributed to these similarities (22). Thus, a definition focusing on similarities between sports potentially dismisses transfers facilitated by other mechanisms.

In the broader literature, talent transfer has also been separated into “product-approach” (talent transfer), or “process-approach” [participation in multiple sports during childhood and adolescence as part of broader development; (73)]. The latter appears to align with the concept of “early diversification” and “sampling” existing in the talent development literature (41). Ambiguous use of terminology, where a term is designated for more than one concept, may compromise the validity of research findings and their applicability to practice. Whilst there may be some commonalities between talent transfer, early diversification, and sampling, they are different concepts with different constructs. This review proposes a holistic definition that moves beyond the narrow view that similarities between sports results in successful transfer and distinguishes it from other pathways and methods of talent identification.

The second objective was to enhance the understanding of the key mechanisms underpinning talent transfer. Athlete factors including anthropometric, physiological, and technical characteristics were prominent amongst the included studies. However, consistent was the finding that these factors alone do not fully capture the complexity of talent transfer. The predominant focus on limited athlete factors for identification into talent transfer programs may lead to similar issues outlined in early talent identification (10, 24). Psychological characteristics were the second most predominant factor, and there have been calls for additional psychological screening for “recruiting and selecting” transfer athletes (24). While certain psychological characteristics can differentiate between elite and non-elite athletes (74), their ability to predict future performance, especially among elite athletes, remains equivocal (9, 75). Unlike other talent identification approaches, talent transfer targets highly trained athletes who likely possess the required psychological attributes. A talent transfer approach, as utilised by Bullock et al. (14) and Hoare & Warr (23), may offer higher predictive validity; identifying a larger cohort of athletes, followed by a talent verification period to select athletes based upon their response to the demands of the new sport, reflective of “representative design” [see (15)]. The inclusion of a talent verification process provides opportunity for a multidisciplinary approach that considers all the factors in talent transfer, as recommended in both research and practice (15, 24, 70).

Initially conceptualised as “mature-age athlete talent identification” (20), this review reveals a broad age range at time of transfer (11–35 years), supporting suggestions that: athlete development models with prescriptive age categories have limited utility for talent transfer [e.g., (41, 44)]; and talent transfer programs that restrict athlete identification based upon age risk deselecting athletes with potential (24). However, this review aggregated data from a diverse range of sports, whereas age of peak performance differs across disciplines and events (76, 77). A systematic review by Allen & Hopkins (76) demonstrated age of peak performance decreases linearly with increasing event duration for explosive power/sprint events and increases linearly with increasing event duration for endurance events. Therefore, to account for difference between sports when designing talent transfer programs, thorough prior analysis is recommended to understand the age profile of the sport. Interestingly, two studies of the same cohort of transfer athletes presented conflicting views on age and its association with high- vs. low-responders (63, 65), highlighting the importance of providing data to substantiate coaches' perspectives on talent and emphasising the need for evidence-based decision-making in talent transfer programs.

Commonalities between environmental factors within talent transfer and the broader talent development literature are evident. Specifically, the importance of micro- (the characteristics of an athlete's immediate environment) and meso- (e.g., sports policies, systems) environmental factors (25, 40, 78), including athlete development processes, coaching, daily training environment, social support, and system factors (16, 41, 79). However, distinction lies in the varied manifestations of these environmental factors in talent transfer. For example, the significance of athlete development processes and the daily training environment is not exclusive to talent transfer [e.g., (40, 78)]; what sets talent transfer apart is the rapid introduction of these processes and environmental attributes, required to accelerate development (14, 23). These inherent differences present unique challenges and opportunities compared to other pathways (21). For instance, in informal occurrences of talent transfer with minimal support from sports, accessing resources such as funding, coaching, and competition can be a barrier to retaining and progressing talented transfer athletes in the recipient sport's pathway (13). Formalising talent transfer pathways and the creation of policies to allocate resources toward talent transfer athletes may offer a solution to address such challenges (21). However, ongoing debate around the legitimacy and effectiveness of talent transfer pathways, coupled with the need for interorganisational collaboration presents unique challenges for sports organisations (21, 24). For sports practitioners and managers, comprehension of these differentiated environmental factors is crucial to optimise talent transfer strategies and policies, thereby promoting overall success.

The third objective was to investigate the application of theory in talent transfer studies. While sports management research has called for theory integration (28, 29), this review demonstrates that talent transfer research lacks a theoretical foundation. Though the FTEM framework offers analytical structure for talent transfer factors, a theoretical understanding of these mechanisms has been lacking (44). This study addresses this gap by utilising ecological dynamics theory to group and appreciate the complexity of the constraints and their interaction within talent transfer (42, 43). This research found the literature has, in a fragmented manner, reported how environmental, task, and individual constraints impact talent transfer (7). Thus, ecological dynamics could be an applicable theory for understanding the mechanisms impacting talent transfer and utilised to enhance future research.

### 4.1 Theoretical implications

There are key theoretical contributions that arise from this systematic review that help advance talent transfer knowledge. This systematic review responds to the need for a more robust definition of talent transfer (13, 21). The review advances the talent transfer definition by consolidating various components from prior definitions into a unified and simplified framework, and considers its boundaries, proposing key determinants, such as the athlete's level as highly trained in their donor sport as crucial for its uniqueness from other talent identification methods (10, 56). Therefore, this paper provides a theoretical step forward by offering a concise and applicable definition of talent transfer, which should provide greater utility in its identification, examination, and practice.

Another key theoretical contribution is emphasising the need for increased use of theory in talent transfer research. Despite longstanding calls for theory integration in sports management research (28, 29), this review reveals talent transfer scholarship lacks a theoretical foundation, particularly for understanding the underlying mechanisms (44). To address this gap, this study draws on ecological dynamics theory to systematically integrate a diverse array of factors and conceptualise how these environmental, task, and individual constraints interact to impact talent transfer (42, 43). This approach could lead to a more consistent and robust theoretical understanding of the multifactorial components of talent transfer, offering insights into its management and facilitation. Furthermore, embracing theories like ecological dynamics may contribute to the holistic development and appreciation of talent transfer scholarship, enhancing the potential for meaningful comparisons between studies, transforming it into a multidisciplinary body of work that incorporates novel managerial perspectives to complement existing sports science- and psychology-based research.

### 4.2 Practical implications

A significant contribution of this review is the comprehensive mapping of factors, which can inform sports practitioners on the development, implementation, and evaluation of talent transfer programs. Through this approach, the present review offers valuable insights into the prevalence of these factors and provides theoretical grounding, facilitating consistent classification both theoretically and operationally. As evidenced throughout the results, studies have often emphasised one influential constraint, rarely considering how they may interact to impact talent transfer. While the inclusion of athlete factors is undeniably important, it is imperative to recognise that these factors alone are insufficient for successful talent transfer. Overreliance on limited athlete factors during the identification stage may replicate shortcomings noted in talent identification and transfer literature (10, 70). Acknowledging the equivocal nature of individual (including psychological) measures in predicting future performance underscores the need for a comprehensive and integrated approach, aligning with a talent verification process and multidisciplinary perspective (15, 70). This holistic process ensures that various individual factors, including psychological characteristics, are considered in response to task and environmental factors, offering a comprehensive understanding of the multifactorial components and likely to enhance the overall success of talent transfer initiatives (10, 15, 24, 70).

### 4.3 Limitations & future research directions

The current review has made some important contributions to the emerging body of talent transfer literature. However, there are some limitations that warrant future research, with some key opportunities in sports management research. First, while the review offers a synthesised definition of talent transfer, further research is required to validate it and ensure its applicability across different contexts (e.g., para-sport vs. non-para-sport, formal vs. informal). Second, while donor and recipient sport organisations and practitioners are inherent to talent transfer (21), there is no research that comprehensively considers the collaboration or conflict between these actors. Strategic sports management research focused on design thinking [e.g., (80)] presents a ripe opportunity to understand how donor and recipient sports stakeholders could work together to design effective talent transfer pathways. Undertaking such research could uncover opportunities for talent transfer, and potential challenges to ensuring its successful implementation.

Third, while meso-level environmental factors are known to be important in sport management [e.g. (81)] and related areas of talent identification and development (6, 79), current research has yet to thoroughly consider or identify how these factors should be promoted in talent transfer. Cury et al. (21) highlighted that sport organisations may benefit from having distinct frameworks that incorporate the provision of funding and performance support for talent transfer athletes. Therefore, future research needs to consider the diversity of meso-environmental factors, such as resourcing, policy, and collaboration between sport organisations, that can be promoted to enhance the success of talent transfer pathways (21).

Another consideration for future research is the potential nuances required for different types of talent transfer. Whilst research often strives for generalisability, it is plausible that different types of talent transfer may need unique theories or conceptualisations. For instance, distinguishing considerations for one-off strategic initiatives, and ongoing talent transfer programs. Para-sport talent transfer may require particularly unique considerations (e.g., athlete classifications and equipment), and scholars suggest that there is a need for para-sport-centric theory to assist pathway management (82). Other types of transitions could also be considered, for example, transferring between individual and team-based sports may provide additional insights as to how pathways need to be managed for talent transfer.

## Data Availability

The original contributions presented in the study are included in the article, further inquiries can be directed to the corresponding author.
